# Chronic Inflammation and Neutrophil Activation as Possible Causes of Joint Diseases in Ballet Dancers

**DOI:** 10.1155/2014/846021

**Published:** 2014-02-20

**Authors:** Leandro da Silva Borges, José Ricardo Bortolon, Vinicius Coneglian Santos, Nivaldo Ribeiro de Moura, Alexandre Dermargos, Maria Fernanda Cury-Boaventura, Renata Gorjão, Tania Cristina Pithon-Curi, Elaine Hatanaka

**Affiliations:** ^1^Instituto de Ciências da Atividade Física e Esportes, Universidade Cruzeiro do Sul, Rua Galvão Bueno 868, 13°Andar, Bloco B, 01506-000 Liberdade, São Paulo, SP, Brazil; ^2^Department of Physiology and Biophysics, Institute of Biomedical Sciences, University of São Paulo, Avenida Lineu Prestes 1524, 05508-900 São Paulo, SP, Brazil

## Abstract

Herein, we investigated the effects of a ballet class on the kinetic profiles of creatine kinase (CK) and lactate dehydrogenase (LDH) activities, cytokines, complement component 3 (C3), and the concentrations of immunoglobulin (Ig), IgA and IgM, in ballerinas. We also verified neutrophil death and ROS release. Blood samples were taken from 13 dancers before, immediately after, and 18 hours after a ballet class. The ballet class increased the plasma activities of CK-total (2.0-fold) immediately after class, while the activities of CK-cardiac muscle (1.0-fold) and LDH (3.0-fold) were observed to increase 18 hours after the class. Levels of the TNF-**α**, IL-1**β**, IgG, and IgA were not affected under the study conditions. The exercise was found to induce neutrophil apoptosis (6.0-fold) 18 hours after the ballet class. Additionally, immediately after the ballet class, the neutrophils from the ballerinas were found to be less responsive to PMA stimulus. *Conclusion*. Ballet class was found to result in inflammation in dancers. The inflammation caused by the ballet class remained for 18 hours after the exercise. These findings are important in preventing the development of chronic lesions that are commonly observed in dancers, such as those with arthritis and synovitis.

## 1. Introduction

Classical ballet is an art that requires daily practice, dedication, and effort to produce light, graceful, and beautiful movements. Dancing has been found to improve motor control, attention, and physical fitness; however, studies have pointed to the intense training as a triggering factor for acute and chronic injuries in dancers [1–3]. Acute and chronic lesions consisting of osteochondral stress, nonstress fractures in the lower extremities and feet, and degenerative arthritis of multiple joints have been reported in ballerinas [4]. The search for perfection by aesthetic standards, coupled with the desire for total control of body movements, compels the dancer to exceed the body's natural limitations, thereby potentially resulting in lesions. Additionally, the body positions in classical ballet are contrary to natural anatomical tendencies and may generate joint misalignments in ballerinas.

Although classical ballet is a widely known and practiced art, there have only been a few limited studies on the physiological and immunological consequences imparted by this kind of dance and the resulting implications on dancers' health. Currently, it is thought that intense exercise accompanied by chronic inflammation may contribute to the appearance of health complications in ballerinas. Studies point to the negative role of inflammation in the progression of inflammatory complications, such as arthritis and arthrosis [5, 6]. Hence, searching for good serum markers of lesions and inflammation that appear during physical exertion may be an important strategy to establish appropriate exercise intensity for ballerinas. Additionally, it is important to establish the amount of time required for ballerinas to recover from exercise-related tissue damage, thereby avoiding chronic inflammation.

Dancing can result in muscle injury, which promotes release of the skeletal muscle enzymes CK and LDH. Both CK and LDH are found in the cytosol; thus, the appearance of these enzymes in the serum indicates muscular lesion [7–9]. The inflammatory response to cellular lesions is initiated by the infiltration of fluid, plasma proteins, and neutrophils into the injured tissue. Cytokines (e.g., TNF-*α*, IL-1*β*, and interleukin-6 [IL-6]) are mediators that participate in the onset of the inflammatory response [10–12]. However, when inflammation and/or strenuous exercise are prolonged, the initial inflammatory response results in the production of hormones related to stress and anti-inflammatory cytokines, such as IL-4, which decrease immunoglobulin (IgA and IgM) concentrations. This may then lead to immunosuppression and a heightened susceptibility to invasive microorganisms, which can negatively affect dancers' performance. Another important point to be considered is the association of exercise-induced muscle damage with neutrophil infiltration into the muscle, thereby resulting in the production of ROS and a systemic elevation in the production of cytokines and other inflammatory mediators and cellular necrosis. Activated neutrophils are a common finding in chronic inflammation [13, 14].

We hypothesized that dancing could expose the dancers to chronic inflammation with reactive neutrophils, which may then lead to the onset of joint problems. This study determined the effects of a ballet class on the levels of TNF-*α*, IL-1*β*, IL-6, IgA, IgG, and C3 in blood samples from the dancers. The proportion of necrotic (loss of plasma membrane integrity) to apoptotic (DNA fragmentation) neutrophils was evaluated. We also analyzed the release of ROS by neutrophils under basal and stimulated conditions. All parameters were assessed before and immediately after the ballet exercise and then 18 hours thereafter.

## 2. Materials and Methods

### 2.1. Materials

RPMI-1640 medium, HEPES, penicillin, and streptomycin were purchased from Invitrogen (Carlsbad, CA, USA). Hydroethidine, Histopaque 1077, PMA (phorbol myristate acetate), LPS (lipopolysaccharide), Triton X-100, and propidium iodide were supplied by Sigma Chemical Co. (St. Louis, MO). Hydroethidine and PMA were dissolved in DMSO (dimethyl sulfoxide). The final concentration of DMSO in the assay medium did not exceed 0.01%. A preliminary experiment showed that DMSO at this concentration is not toxic for neutrophils and does not interfere in the results obtained [15]. Reagents, water, and plastic wares used in the experiments were all endotoxin-free.

### 2.2. Subjects

With the approval of the Ethics Committee of Cruzeiro do Sul University (115/2009), 13 women volunteers participated in the study. All of the ballerinas signed an informed consent form agreeing to submit to the procedures involved in the study. The group had the following characteristics (mean ± SE): age 20.0 ± 0.8 years, body mass 41.4 ± 1.6 kg, height 1.6 ± 0.0 m, body fat 24.0 ± 1.3%, VO_2peak_ 36.8 ± 2.9 mL·kg^−1^·min^−1^, and sports experience of 7.0 ± 1.5 years. The ballerinas used to dance 4–6 hours, five days of the week and rest in the weekend. The participants had rested for 72 hours before the ballet class. All the participants danced for 70 minutes, according to a protocol established by Guidetti et al. [16].

The analysis of the ballet dancers' physical and metabolic profiles through the assessments of blood pressure, heart rate, blood glucose, and muscle strength testing revealed no differences in the dancers before, immediately after, and 18 hours after a ballet class (data not shown). Participants with histories of infection, viruses, chronic lesions, diabetes, rheumatoid arthritis, hormonal dysfunction, lupus, or other inflammatory and hematology diseases (such as hemoglobinopathies) and who were taking medication were excluded from the study.

### 2.3. Sample Collection

Twenty milliliters of venous blood was collected before, immediately after, and 18 hours after a ballet class. The blood samples were drawn from one of three main veins at the antecubital fossa (the cephalic, basilic, and median cubitals). In each case, the vein was chosen based on the identification of an optimal site by both visual and tactile exploration. The blood samples were drawn into two BD vacutainer tubes, with the first containing heparin, which was used for plasma collection and cell separation; the second was a dry gel tube for serum collection. After collecting the samples, the blood was centrifuged (400 ×g, 10 minutes), and the serum and plasma were separated from the cell components. Neutrophils were immediately isolated, and cellular function was tested. The enzymatic activities of CK and LDH were measured not later than 48 hours after the collection of the plasma. Whenever necessary, the samples were diluted to fall within the linear range of the methods. All of the dilutions were performed with suitable dilution reagents, according to the instructions of the kit manufacturer (DuoSet Kit: Quantikine, R&D System, Minneapolis, MN, USA). The concentrations of diluted samples were determined after multiplying the reported values by the dilution factor.

Plasma was collected and stored at −80°C prior to cytokine determination by ELISA. The samples were stored for no longer than 3 months.

### 2.4. Determination of Creatine Kinase (CK) and Lactate Dehydrogenase (LDH) Activities

Serum CK and LDH activities were measured according to the methods established by Zammit and Newsholme [17]. The kits were supplied by Bioclin Diagnostics (São Paulo, SP, Brazil), and the measurements were performed according to the manufacturer's instructions. The control serum was used to check the accuracy and precision of the assay, with a maximum error of 5%.

### 2.5. Determination of Plasma Cytokines

Plasma levels of IL-1*β*, TNF-*α*, IL-6, and IL-10 were determined by ELISA, according to the manufacturer's instructions (DuoSet Kit: Quantikine, R&D System, Minneapolis, MN, USA). The IL-6 and IL-10 methods were linear for protein concentrations in the ranges of 25 picograms per milliliter (pg/mL) to 600 pg/mL and 25 pg/mL to 2000 pg/mL, respectively. The TNF-*α* method was considered to be linear for protein concentrations in the range of 6.0 pg/mL to 1000 pg/mL; for IL-*β*, the range was 5.0 pg/mL to 250 pg/mL. A standard curve was built for each set of samples, and the cytokines were assayed, yielding a correlation coefficient in the range of 0.98 to 0.99. For these determinations, the intra-assay coefficient of variance was 3–5%, while the interassay coefficient of variance was 8–10%.

### 2.6. Cell Purification

The experiments were performed within 1 h of venipuncture. Human neutrophil (>98%) preparations were isolated from the peripheral blood of human donors under endotoxin-free conditions using Histopaque 1077 (Sigma Chemical Co., St. Louis, MO) according to the manufacturer's instructions. Briefly, blood was diluted v/v with 10 mM PBS Dulbecco at pH 7.4 and carefully layered on 10 mL of a commercial gradient of Ficoll-Hypaque (Histopaque, *d* = 1.077). The tube was centrifuged at 400 g at room temperature for 20 min. The supernatant, rich in mononuclear cells, was discarded, and 10 mL of 5% dextran was added to the pellet. The tube was homogenized and maintained for 45 min on ice to allow erythrocyte sedimentation. The resulting supernatant, rich in granulocytes, was recovered, washed with PBS Dulbecco, and the pellet submitted to hypotonic treatment with 10 mL of distilled water to promote lysis of contaminated erythrocytes. After 1 min, the isotonicity was restored by the addition of 5 mL of 2.7% NaCl and 15 mL of PBS Dulbecco. Cells were centrifuged at 400 g for 5 min at room temperature and suspended in RPMI 1640 medium. The purity of the cell preparation was higher than 98%.

### 2.7. Cell Viability Assay (Proportion of Necrotic Cells)

Neutrophil viability was assessed using a FACSCalibur Cytometer (Becton Dickinson Systems, CA, USA). The percentage of viable cells in each sample was determined based on propidium iodide staining (0.05% w/v solution in PBS). Ten thousand events were analyzed per sample. Fluorescence of the propidium iodide was measured using the FL2 channel (orange-red fluorescence = 585/545 nm) [18].

### 2.8. Proportion of Cells with DNA Fragmentation

DNA fragmentation was analyzed by flow cytometry after DNA staining with propidium iodide (Sigma Chemical Co., St. Louis, MO). The presence of detergent in the solution permeabilized the cells, which promptly incorporated the dye into DNA. After incubation, the cells were centrifuged at 1000 ×g for 15 min at 4°C. The resulting pellets were carefully resuspended in 300 *μ*L hypotonic solution containing 50 *μ*g/mL propidium iodide, 0.1% sodium citrate, and 0.1% Triton X-100. The cells were then incubated for 30 min at 4°C. Ten thousand events were analyzed per sample. Fluorescence of the propidium iodide was measured using the FL2 channel (orange-red fluorescence = 585/545 nm) [18].

### 2.9. Flow Cytometric Measurement of Reactive Oxygen Metabolites Using Hydroethidine

Hydroethidine (1 *μ*M) was added to the neutrophil (2.5 × 10^6^ cells/mL) incubation medium when required. Immediately afterwards, the cells were treated with PMA (54 ng/mL). ROS release was monitored for 30 minutes. The assays were run in PBS buffer supplemented with CaCl_2_ (1 mM), MgCl_2_ (1.5 mM), and glucose (10 mM) at 37°C in a final volume of 0.3 mL. Hydroethidine (HE) has been widely used for the flow cytometric measurement of intracellular ROS production. Hydroethidine, a reduced derivative of ethidium bromide, easily penetrates into the cells and shows weak fluorescence weakly when excited by light at 480 nm wavelength. Hydroethidine is intracellularly oxidised by oxygen radicals, being converted into ethidium bromide that tightly binds to DNA and shows a strong red fluorescence that was measured using the FL3 channel of a FACSCalibur flow cytometer (Becton Dickinson, CA, USA). Ten thousand events were analyzed per experiment [15, 18].

### 2.10. Externalization of Phosphatidylserine

The externalization of phosphatidylserine was analysed by flow cytometry after PS staining with annexin V-FITC. Annexin V is a phospholipid-binding protein that has a high affinity for PS. PI is used to distinguish viable from nonviable cells. Fluorescence of annexin V-FITC was measured as described above in FL1 channel (green fluorescence; 530/30 nm) and PI in FL2 channel (orange/red fluorescence; 585/545 nm).

### 2.11. Determination of the MTP (Mitochondrial Transmembrane Potential)

Cells were centrifuged at 1000 g for 10 min at 4°C, and the pellet was resuspended in 500 *μ*L of PBS. Rhodamine 123 (Sigma Chemical Co., St. Louis, MO) is a cell-permeant cationic fluorescent dye that is readily sequestered by active mitochondria without inducing cytotoxic effects. Rhodamine 123 (5 *μ*mol/L) was added and the cells were then incubated for 15 min in the dark. Cells were washed twice with ice-cold PBS and incubated for 30 min in the dark. Fluorescence was determined by flow cytometry using the FL1 channel (green fluorescence; 530/30 nm) as described above.

### 2.12. Statistical Analysis

The values are presented as the means ± standard errors of 13 ballerinas. The statistical analysis consisted of one-way analysis of variance (ANOVA) using the post-hoc Student-Newman-Keuls Multiple Comparison test (INStat; Graph Pad Software, San Diego, CA, USA). The significance level was set at *P* < 0.05. The degree of linear relationship between CK and LDH variables was established by Pearson's correlation.

## 3. Results

### 3.1. Effect of a Classical Ballet Class on Markers of Muscle Damage

**Figure 1:**
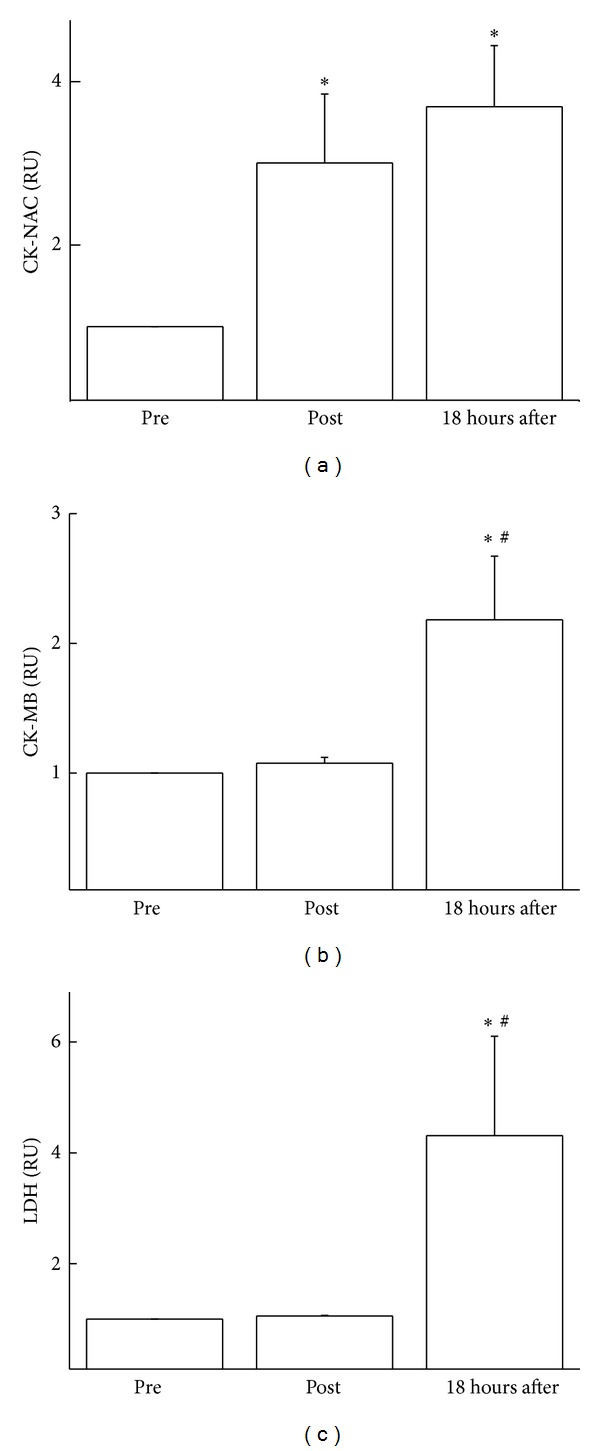
Effect of classical ballet on muscle lesion markers. Serum concentrations of CK-NAC (a), CK-MB (b), and LDH (c) in dancers were determined before, immediately after, and 18 hours after a ballet class. The values are presented as mean ± standard error of 13 dancers and are presented as relative unit (UR). Figures were plotted with the data of the variations in markers during a ballet class, thus indicating the normalization between the values of before and after a ballet class. *P < 0.05 for comparison to preclass condition and ^#^ *P* < 0.05 for comparison to immediately after ballet class condition.

Figure 1 shows the variations of CK-NAC, CK-MB, and LDH in the blood of ballet dancers immediately after and 18 hours following a classical ballet class. We observed a significant increase in the activity of CK-NAC (2.0-fold, *P* < 0.05) immediately after and 18 hours after the ballet class (2.0-fold, *P* < 0.05) (Figure 1(a)). Plasma activities of CK-MB (1.0-fold, *P* < 0.05) (Figure 1(b)) and LDH (3.0-fold, *P* < 0.05) (Figure 1(c)) were also observed to increase in the 18-hour period following the class. Figures 1(a), 1(b), and 1(c) were plotted with the data of the variations in markers during a ballet class, thus indicating the normalization between the values of before and after a ballet class. The values are presented as relative unit (RU).

### 3.2. Effect of a Classical Ballet Class on Inflammatory Markers

Under the study conditions, a decrease in the concentration of IL-4 (3.0-fold, *P* < 0.05) was also noted 18 hours after the class (Table 1). No significant differences were identified in the blood concentrations of IL-1*β*, TNF-*α*, IL-6, IL-10, IL-2, C3, IgA, and IgG (Table 1).

### 3.3. Effect of a Classical Ballet Class on Death and Functionality of Neutrophils

**Figure 2:**
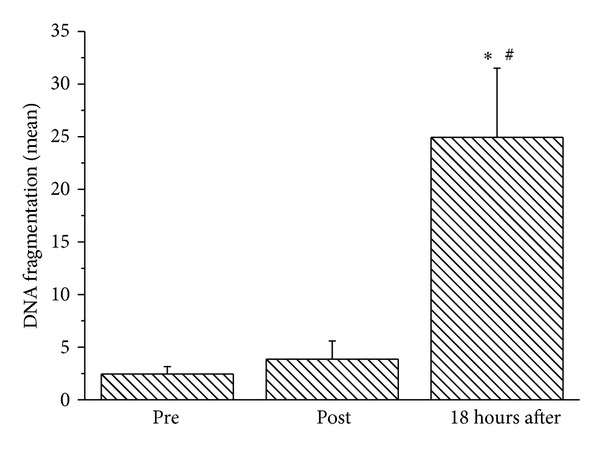
Neutrophil DNA fragmentation (%) in ballet dancers determined before, immediately after, and 18 hours after a ballet class. The values are presented as the mean ± standard error of 13 dancers. *P < 0.05 for comparison to preclass and ^#^ *P* < 0.05 for comparison to immediately after ballet class.

We noted a significant increase in the mean number of neutrophils with fragmented DNA at 18 hours after the class (6-fold, *P* < 0.05) (Figure 2). The neutrophil analysis did not reveal any significant changes in the number of cells, cell membrane integrity, externalization of phosphatidylserine, or mitochondrial depolarization (Table 2).

**Figure 3:**
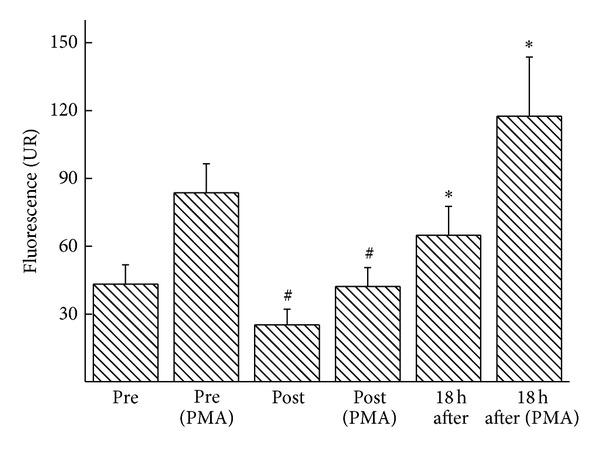
ROS release by neutrophils in ballet dancers was determined before, immediately after, and 18 hours after a ballet class. The measurements were performed under basal and PMA-stimulated conditions. The values are presented as the means ± standard errors of 13 dancers.

As shown in Figure 3, we identified changes in neutrophil function from ballerinas immediately following a classical ballet class and found that neutrophils were less responsive to PMA stimuli.

## 4. Discussion

A number of factors are thought to contribute to injuries among ballerinas, including the young age at which intensive dance training begins, dancing *en pointe*, antianatomical body positions, unusual dietary regimens, and emotional stress. In fact, in ballerinas, the most important cause of increased arthritis and arthrosis may be partially explained by repetitive microtrauma that results in chronic inflammation [19, 20]. Thus, prevention studies are required to reduce the incidence, severity and cost of ballerina injuries. Moreover, while dance injuries have been the target of numerous studies, we have not identified any studies in the literature describing the relationship of injuries to the immune system that specifically relate ballet lesions, inflammation, and biochemical/immunological markers.

Our results demonstrated that a simple ballet class increased the plasma activities of the muscle enzymes CK and LDH and decreased the concentration of the anti-inflammatory cytokine IL-4. In addition, 18 hours after the class, signs of muscular lesions, neutrophil necrosis, and decreased levels of anti-inflammatory cytokines all pointed to a subclinical inflammatory condition. TNF-*α*, IL-1*β*, IgG, and IgA blood levels were not affected in the conditions studied.

One day following a classical ballet class, the ballerinas exhibited a significant increase in the mean number of neutrophils with fragmented DNA. Apoptosis is now known as an active cell death process that is characterized by the activation of proteases, autodestruction of chromatin (DNA fragmentation), nuclear condensation, cellular membrane blebbing, and vascularization of internal components. Our group has demonstrated that professional athletes participating in an adventure race [21] and triathlon present leukocyte death after the competition [22, 23]. While the stress associated with acute exercise has been reported to induce significant leukocyte apoptosis, not all investigations have confirmed this finding, and the mechanism behind exercise-induced apoptosis remains unclear. Some hypotheses suggest the following: (i) the elimination of excessive cells that appear during exercise is a physiological response; (ii) normal regulatory processes that serve to remove certain damaged cells do so without a pronounced inflammatory response; (iii) apoptosis is the body's response to excessive oxidative stress; (iv) increases in cytokines such as TNF-*α* serve as important signaling molecules in apoptotic and other signaling pathways [24, 25].

Neutrophils are the first cells to migrate to the site of injury and are responsible for local cleaning, phagocytosing pathogens, and removing cellular debris. The initial release of proinflammatory mediators and ROS and the activation of neutrophils are important events of tissue repair; however, the inflammatory response must be a self-controlled event [26–29]. The hypoproduction of ROS by neutrophils that come into contact with stimuli may lead to increased susceptibility to invasive microorganisms, thereby impairing the dancers' performance and health. In these aspects, our results demonstrated that neutrophils are less responsive to PMA stimuli immediately after a ballet class. These results point to the reduced efficiency of neutrophils against infection when exposed to pathogens immediately after a ballet class.

**Figure 4:**
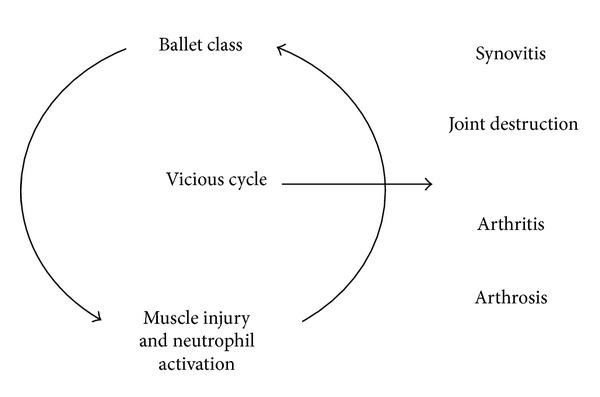
Chronic inflammations as a result of ballet dancing class causes subclinical systemic inflammation and the activation of the function of neutrophils in the chronic inflammation may be the main factor involved in the genesis of arthritis and other inflammatory joint diseases in ballerinas.

Chronic inflammation is characterized by influxes of neutrophils at sites of inflammation and may be the main factor involved in the development of arthritis, arthrosis, and other inflammatory joint diseases that have been observed in ballerinas (Figure 4). Experimental data indicates that neutrophils play an important role in both the immunization and the effector phases of autoimmune diseases, such as arthritis. The fact that neutrophils are involved in autoantibody-induced arthritis indicates that these cells contribute to the autoantibody-mediated component of the disease's effector phase. Neutrophils appear to be required for the extravasation and deposition of autoantibodies at the joints [30].

Other neutrophil analyses demonstrated no significant changes in the number of cells, cell membrane integrity, externalization of phosphatidylserine, and mitochondrial depolarization.

The mechanical stressors on joints arise from many sources, including misalignments of bones caused by antianatomical ballet positions, mechanical injury, overtraining, or jumps that overstress joints and result in local inflammation. Chronic inflammation as a result of a nonperiodized resting period results in constant activation of the immune system and may be dangerous for the dancers' health.

## 5. Conclusions

In summary, we have found that immediately after a ballet class, neutrophils from ballerinas were found to be less responsive to stimuli, thereby pointing to a transient immunosuppression. Eighteen hours after the class, signs of muscular lesion, neutrophil necrosis, and decreased levels of anti-inflammatory cytokines were observed in the ballerinas, thereby suggesting a persistent inflammatory condition. These findings may represent an extremely useful tool to design new studies and strategies to protect dancers against microorganism infection as a result of transient immunosuppression immediately following the class and to prevent dancers performance from declining as a result of chronic inflammation.

## Figures and Tables

**Table 1:** Effects of classical ballet class on the serum concentrations of IL-1*β*, TNF-*α*, IL-6, IL-10, IL-2, IL-4 and C3 were quantified in the plasma, and serum concentrations of IgA and IgG were quantified in the serum of dancers before, immediately after and 18 hours after a ballet class.

	Before	After	18 hours after
IL-1*β* (ng/mL)	0.29 ± 0.08	0.25 ± 0.06	0.23 ± 0.06
TNF-*α* (ng/mL)	0.16 ± 0.04	0.15 ± 0.04	0.12 ± 0.01
IL-6 (ng/mL)	0.11 ± 0.03	0.13 ± 0.03	0.08 ± 0.02
IL-10 (ng/mL)	0.12 ± 0.01	0.12 ± 0.01	0.13 ± 0.01
IL-2 (ng/mL)	0.04 ± 0.005	0.04 ± 0.005	0.02 ± 0.02
IL-4 (ng/mL)	88.00 ± 1.00	86.85 ± 1.93	81.74 ± 0.74*
IgA (mg/dL)	198 ± 13	185 ± 17	196 ± 13
IgG (mg/dL)	27 ± 3	29 ± 3	33 ± 3
C3 (mg/dL)	219 ± 0.87	218 ± 2.00	219 ± 2.55

The values are presented as the means ± standard errors of 13 dancers. mg/dL: milligrams per deciliter. *P < 0.05 for comparison to pre-class conditions.

**Table 2:** Effects of a classical ballet class on neutrophil numbers, membrane integrity, externalization of phosphatidylserine and mitochondrial depolarization before, immediately after and 18 hours after a ballet class.

Neutrophils	Before	After	18 hours after
Number of Neutrophils (×10^6^)	41.83 ± 8.89	48.5 ± 19.72	26.66 ± 8.38
Cell Membrane Integrity (mean)	63.95 ± 14.21	53.92 ± 15.07	53.70 ± 14.32
Externalization of Phosphatidylserine (mean)	11.14 ± 3.30	9.77 ± 4.31	6.17 ± 2.58
Mitochondrial Depolarization (mean)	114.94 ± 11.31	121.50 ± 11.69	155.23 ± 31.62

The values are presented as the means ± standard errors of 13 dancers. *P < 0.05 for comparison to pre-class conditions.
